# Disruption of Steroid Axis, a New Paradigm for Molar Incisor Hypomineralization (MIH)

**DOI:** 10.3389/fphys.2017.00343

**Published:** 2017-05-26

**Authors:** Sylvie Babajko, Katia Jedeon, Sophia Houari, Sophia Loiodice, Ariane Berdal

**Affiliations:** ^1^Laboratory of Molecular Oral Pathophysiology, Centre de Recherche des Cordeliers, Institut National de la Santé et de la Recherche Médicale UMRS 1138, University Paris-Descartes, University Pierre et Marie Curie-ParisParis, France; ^2^Unité de Formation et de Recherche en Odontologie, University Paris-DiderotParis, France; ^3^Centre de Référence des Maladies Rares de la face et de la Cavité Buccale MAFACE, Rothschild HospitalParis, France

**Keywords:** amelogenesis, MIH, steroid receptors, steroid hormones, endocrine disrupting chemicals, enamel mineralization

## Overview

Molar-Incisor Hypomineralization (MIH) is a common developmental enamel defect characterized by asymmetric demarcated opacities in permanent molars and incisors. MIH was first described in 2001–2003 (Weerheijm et al., 2001; Weerheijm and Merjare, 2003). It was previously called cheese molars, idiopathic enamel hypomineralization in permanent teeth, included in developmental enamel defects other than that caused by fluoride but the prevalence of these defects was poorly documented except in Sweden where it was first investigated (Koch et al., 1987). It affects now 15–20% of 6–9 year-old children worldwide but its etiology still remains unclear. MIH is certainly a non-hereditary multifactorial pathology even though an individual hereditary susceptibility to MIH is not excluded as suggested by enamelin gene polymorphism (Jeremias et al., 2013). Several causal factors have been proposed such as prematurity, long breastfeeding, viral or bacterial infections, respiratory diseases, asthma (Alaluusua et al., 2002; Alaluusua, 2010; Serna et al., 2016; Silva et al., 2016; Tourino et al., 2016). None of these factors is satisfactory to explain MIH recent emergence nor its selective enamel lesions on the first mineralizing permanent teeth, mainly permanent first molars and incisors. Despite the fact that mineralization of the other permanent teeth may be delayed, they are rarely affected by MIH. Given that MIH affects those teeth undergoing mineralization around the time of birth, it is clear that the enamel forming ameloblasts are sensitive to the causative agent(s) in a specific time window only. It is noteworthy that MIH emergence is overlaying to increased prevalence of pathologies related to the currently changing environmental conditions with increasing amounts of pollutants. Indeed, our environment and lifestyle are dramatically changing and exposure to novel molecules or combination of factors during the period of amelogenesis may be a possible track. Among environmental toxicants, Endocrine Disrupting Chemicals (EDCs) are exogenous substances or mixtures that alter function(s) of the endocrine system and consequently cause adverse health effects in an intact organism, or its progeny, or (sub) populations (EDC definition established by the World Health organization in 2002). EDCs are small molecules that may share structural homologies with steroid hormones, and are thus able to disrupt steroid axes. Steroid hormones (such as estrogens, androgens, or corticoids for example) mediate their effects through intracellular steroid receptors that modulate transcription of their target genes. Most of steroid receptors are expressed by ameloblasts and thus possibly involved in amelogenesis (Houari et al., 2016). The present paper explores the hypothesis of their involvement in amelogenesis and delineates one mechanistic path that would account for MIH.

## Evidence

EDCs have often been proposed to contribute to hormone-dependent cancers, decreased fertility, diabetes, obesity, and cognitive disorders over the past 50 years (Gore et al., 2015). This hypothesis is supported by a number of recent epidemiological and experimental studies. Among the thousands of EDCs, bisphenol A (BPA) is one of the most active and widely used by the plastic industry and also for dental materials. It may be leached as an active monomer under several conditions (Cooper et al., 2011). Sensitivity to BPA is the greatest during the perinatal period and many pathologies diagnosed during adulthood would result from fetal and perinatal exposure to these molecules (Poimenova et al., 2010; Varayoud et al., 2014; Braun, 2017). Interestingly, this period of time corresponds to the temporal window when the enamel of the human permanent teeth is being formed.

Our recent data showed that human MIH and BPA exposed rat teeth present similar structural and biochemical characteristics (Jedeon et al., 2013). Both series of teeth present broken enamel in areas where the teeth occlude. In addition, the prismatic structure in human MIH enamel as well as BPA exposed rat enamel was obscured by a covering organic layer (Jedeon et al., 2013) similar to the one reported previously (Jälevik et al., 2005). Among the main enamel matrix proteins, enamelin expression was higher in BPA exposed ameloblasts. Enamelin amount is a central parameter for enamel synthesis as demonstrated by an experimental genetic approach (Hu et al., 2014). Indeed, *ENAM* mutations have been reported in Amelogenesis Imperfecta (AI) (Lindemeyer et al., 2010; Chan et al., 2011), and have been associated with MIH (Jeremias et al., 2013). Specific alleles of *ENAM* are also associated with high susceptibility to dental caries (Chaussain et al., 2014) and the expression level of enamelin appears to be determinant for the structure and quality of enamel (Hu et al., 2014). Too much or too little enamelin abolishes the formation of enamel crystals and prism structure. BPA has also been shown to decrease KLK4 expression which is involved in the degradation of enamel matrix proteins (Jedeon et al., 2013). KLK4 is a serine-protease that cleaves enamel matrix proteins to permit enamel full and correct enamel mineralization (Bartlett and Simmer, 2014). *KLK4* mutations have also been reported in AI (Chan et al., 2011). When KLK4 activity and/or level of expression is reduced, remaining enamel proteins after the maturation process of enamel inhibit normal apatite crystal growth. This second event strengthens the first one by additionally increasing the amount of remaining enamelin in mature enamel. In such case, extraneous proteins such as serum albumin are able to accumulate in the poor quality enamel (Farah et al., 2010) worsening the hypomineralization, finally diagnosed as white opaque spots (Denis et al., 2013).

Human and animal populations are exposed to many EDCs simultaneously. BPA certainly acts in combination with other EDCs or hypomineralizing agents. These molecules do not necessarily share the same structural properties, and act through different signaling pathways and receptors. Consequently, the effects of EDCs combinations are unpredictable. For example, combination of low doses of BPA with low doses of genistein and vinclozolin, two other EDCs, didn't lead to a greater phenotype (Jedeon et al., 2014a) whereas combination of BPA with fluoride increased enamel hypomineralization (Jedeon et al., 2016a). Enamel defects have also been associated to exposure to dioxin (Alaluusua et al., 2004) and PCBs (Jan et al., 2007), two groups of pollutants presenting EDC activity. Interestingly, dioxin and amoxicillin exposures have been proposed as a causal factor of Molar Incisor Hypomineralisation (MIH) (Alaluusua et al., 1999; Laisi et al., 2009). It is noteworthy that both factors increase enamel hypomineralization in the presence of fluoride (Salmela et al., 2011; Sahlberg et al., 2013) and the importance of the perinatal exposure to these agents has been underlined (Alaluusua et al., 2002). Even if fluoride is probably not a causal factor of MIH, experimental fluoride in combination with EDCs was shown to increase enamel hypomineralization (Salmela et al., 2011; Sahlberg et al., 2013; Jedeon et al., 2016a).

**Figure 1:**
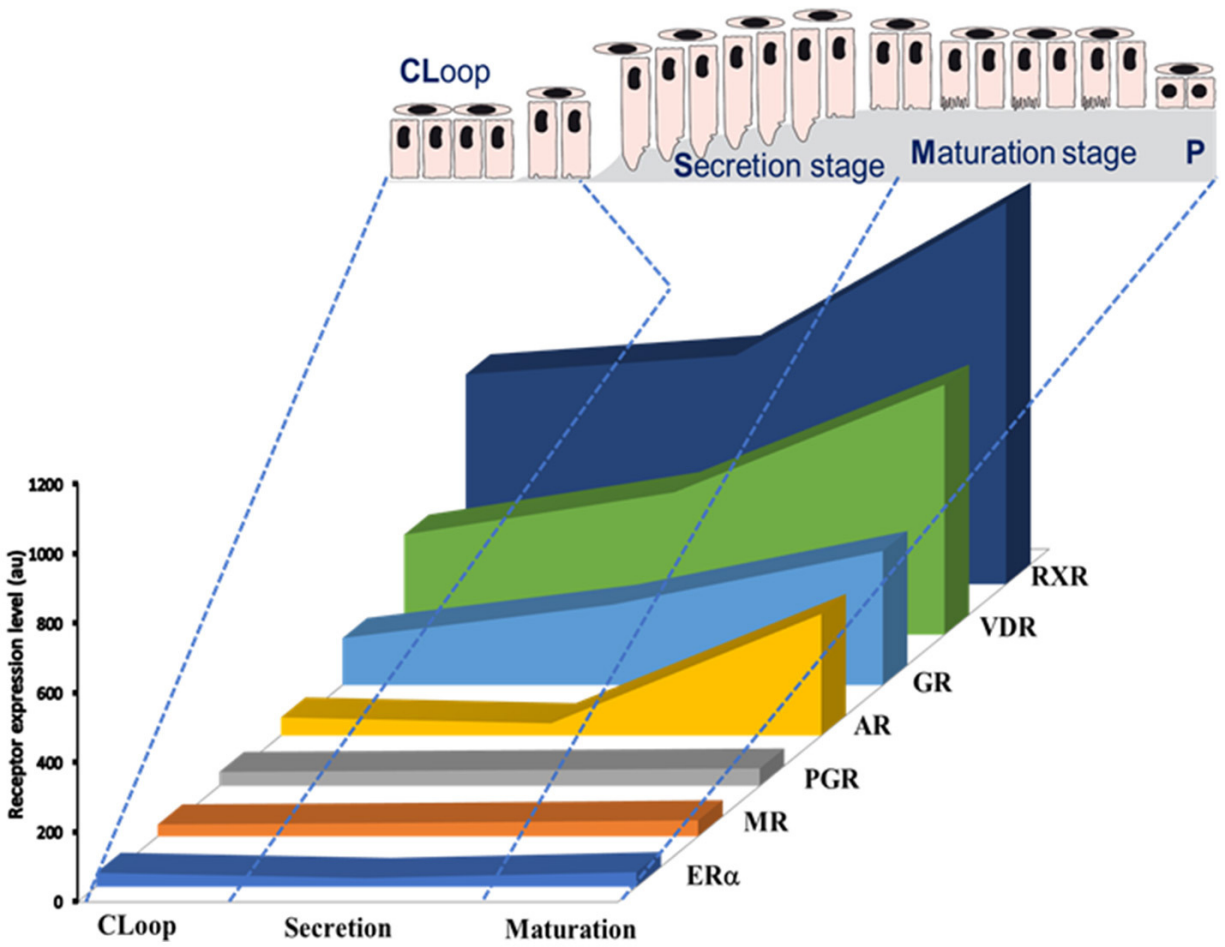
**Schematic representation of expression profiles of steroid receptors during amelogenesis based on data published by Houari et al. (2016)**. The relative level of expression of each mRNA was determined by microarray analysis of RNAs extracted from maturation-stage enamel organ.

A number of EDCs are known to disrupt the steroid axis. BPA, for example, binds ERs (Delfosse et al., 2012), GPR30 (Pupo et al., 2012), and ERRγ with high affinity (Liu et al., 2012; abbreviations in Table 1). BPA is also able to, directly or indirectly, modulate the activity of AR, PR, GR, RXR, and PPARγ receptors (Li et al., 2015; Rehan et al., 2015). Except PPARγ and ERβ, rodent ameloblasts express all these receptors and their expression levels vary depending on the ameloblast differentiation stage (Houari et al., 2016; Figure 1). Furthermore, we have shown that ERα is involved in pre-ameloblast proliferation (Jedeon et al., 2014b), and AR in the enamel terminal mineralization process (Jedeon et al., 2016b). Thus, mediated by these receptors, EDCs such as BPA and vinclozolin may disrupt amelogenesis. GR and VDR are classically associated to amelogenesis and enamel mineralization (Pawlicki et al., 1992; Berdal et al., 1993) and might also play a role in the transmission of EDC effects.

**Table 1:** **List of abbreviations cited in the text**.

AhR	Aryl hydrocarbon Receptor
AI	Amelogenesis Imperfecta
AR	Androgen Receptor
BPA	Bisphenol A
EDC	Endocrine Disrupting Chemical
ER	Estrogen Receptor
ERRγ	Estrogen Related Receptor γ
GPR30	G-Protein-Coupled Receptor 30
GR	Glucocorticoid Receptor
KLK4	Kallikrein-related peptidase 4
MIH	Molar Incisor Hypomineralization
PCB	PolyChlorinated Biphenyl
PPARγ	Peroxisome Proliferator-Activated Receptor γ
PR	Prolactin Receptor
RAR	Retinoic Acid Receptor
RXR	Retinoid X Receptor
VDR	Vitamin D receptor

All these data argue for the steroid axis playing a central role in the physiological as well as pathological process of amelogenesis. The presence of these receptors which expression vary during amelogenesis suggests a stage-specific susceptibility to the corresponding ligands. These may be endogenous molecules like hormones, or exogenous such as vitamins, drugs and EDCs. Otherwise, data reported in the literature showed that many if not all MIH causal factors hypothesized are associated, directly or indirectly, with steroid axis:

Indeed, prematurity and long breastfeeding have been associated to MIH but seem controversial (Alaluusua, 2010; Sönmez et al., 2013). If so, it's worthy to note that milk may accumulate pollutants such as dioxin and PCBs, acting through AhR sharing signaling pathway with ERs (Solomon and Weiss, 2002). On the other hand, premature babies were reported to be contaminated with BPA and phthalates essentially due to medical devices (Calafat et al., 2009; Duty et al., 2013). And, both class of EDCs act via steroid receptors, ERs and AR, reported to modulate enamel key genes like KLK4 (Jedeon et al., 2016b).

MIH is also associated to infections, otitis, bronchitis, pneumonia, fever and asthma (Tourino et al., 2016). These pathologies are often treated with antibiotics combined to anti-inflammatory molecules as corticoids, acting through GR, which may lead to enamel hypomineralization. There are typical responsive elements to GR in the amelogenin promoter which is a key component of enamel matrix (Gibson et al., 1997) and exposure to corticoids was associated to enamel hypomineralization in rats (Pawlicki et al., 1992).

Deficiency in vitamin A acting through RAR/RXR pathway has been recently associated to MIH (Mishra and Pandey, 2016). Ameloblasts express retinoid receptors and binding proteins (Bloch-Zupan et al., 1994; Houari et al., 2016) and excess of retinoids disrupt amelogenesis leading to enamel hypomineralization (Morkmued et al., 2017), meaning that the right concentration of retinoids is required at the right moment of amelogenesis. Another vitamin which deficiency was associated to MIH is vitamin D (Kühnisch et al., 2015). It is well-known that vitamin D binds to the heterodimer VDR/RXR which are the most highly expressed steroid receptors in maturation-stage ameloblasts (Figure 1). Vitamin D and VDR are tightly associated to enamel mineralization (Berdal et al., 1993). And, the steady-state mRNA levels of enamel matrix peptides were shown vitamin-dependant in vitamin D deficient rats which harbored malformed enamel (Papagerakis et al., 2002). In addition, levels of vitamin D were inversely correlated to BPA contamination (Johns et al., 2016) suggesting a protective role of vitamin D against EDC adverse effects and reinforcing the idea of the importance of steroid axis during the pathophysiology of amelogenesis.

## Conclusion

Many of the proposed causal factors for MIH, including EDCs, anti-inflammatory corticoids, vitamin deficiency involve the large family of the steroid receptors. Most of the steroid receptors are expressed in ameloblasts and their levels of expression are dependent on their stage of differentiation. The steroid receptors thus appear as the common elements able to modulate the expression of enamel key genes controlling enamel synthesis or leading to enamel hypomineralization in case of disruption.

## Author contributions

SB raised the hypothesis of the paper, drafted and wrote the paper. KJ, SH, and SL did experiments, obtained the results and wrote the corresponding published papers cited in the text. AB drafted, read the paper and made helpful suggestions to improve the paper. All authors approved the final version to be published.

### Conflict of interest statement

The authors declare that the research was conducted in the absence of any commercial or financial relationships that could be construed as a potential conflict of interest.
